# Physiological functions of urea transporter B

**DOI:** 10.1007/s00424-019-02323-x

**Published:** 2019-11-22

**Authors:** Lanying Yu, Tiantian Liu, Shuang Fu, Li Li, Xiaoping Meng, Xin Su, Zhanfeng Xie, Jiayan Ren, Yan Meng, Xuejiao Lv, Yanwei Du

**Affiliations:** 1 Changchun University of Chinese Medicine, Changchun, 130117 People’s Republic of China; 2 Department of Pathophysiology, College of Basic Medicine, Jilin University, Changchun, 130021 Jilin, People’s Republic of China; 3 Department of Respiratory Medicine, the Second Affiliated Hospital of Jilin University, Changchun, 130041 Jilin, People’s Republic of China

**Keywords:** Urea, Urea transporter proteins, UT-B, Kidd blood group, UT-B-null mice

## Abstract

Urea transporters (UTs) are membrane proteins in the urea transporter protein A (UT-A) and urea transporter protein B (UT-B) families. UT-B is mainly expressed in endothelial cell membrane of the renal medulla and in other tissues, including the brain, heart, pancreas, colon, bladder, bone marrow, and cochlea. UT-B is responsible for the maintenance of urea concentration, male reproductive function, blood pressure, bone metabolism, and brain astrocyte and cardiac functions. Its deficiency and dysfunction contribute to the pathogenesis of many diseases. Actually, UT-B deficiency increases the sensitivity of bladder epithelial cells to apoptosis triggers in mice and UT-B-null mice develop II-III atrioventricular block and depression. The expression of UT-B in the rumen of cow and sheep may participate in digestive function. However, there is no systemic review to discuss the UT-B functions. Here, we update research approaches to understanding the functions of UT-B.

## Introduction

Urea is an organic compound with two NH2 groups linked by a carbonyl. Urea has high water-solubility and low fat-solubility. Urea is produced in the liver and concentrates in the kidney for nitrogen excretion in mammals. Although urea is not charged, it has a strong dipole moment, which makes it impossible to penetrate through non-polar lipid membranes. Hence, urea depends on its specific transporters to pass through the cell membranes.

At present, some genes for urea transporters (UTs) have been cloned from human, rabbit, mice, and Xenopus, and they include the SLC14a1 gene for two subtypes of UT-Bs and the SLC14a2 gene for six subtypes of UT-As due to varying splicing. Here, we review the gene, protein structure, and main functions of UT-B in different organs.

## Urea transporters

UT proteins were discovered in the kidney medullary collecting ducts of rabbits and mice, and the SLC14a1 for UT-B (B1, B2) was cloned from the renal medulla of rabbits and from human bone marrow [19, 55]. Subsequently, the SLC14a2 gene for UT-A (A1–A6) was cloned from human, rabbit, mice, and Xenopus, and UT-A1, UT-A2, and UT-A3 are sensitive to vasopressin. These genes are located in human chromosome 18 q12.1-q21.2 [31, 54, 66]. The UT-B gene has 11 exons, of which exons 4–11 contain the coding sequence for the UT-B of 30 kDa. UTs are widely expressed in mammals. While the UT-A is expressed mainly in human kidney and other organs, including the testis, colon, and liver, the UT-B is highly expressed in human erythrocytes and many other organs (Table 1) [70]. Urea transporters are N-linked glycosylated proteins with a unique hydrophobic character. Human UT-B1 is a glycosylated protein of 46–60 kDa in most organs, except for a 41–54-kDa protein in the kidney, but it is deglycosylated as a 36-kDa protein in red blood cells. Similar tissue-specific variates in the degrees of glycosylation are observed in other species. For example, the rat glycosylated UT-B1 protein is 32 kDa in the brain while it is 45–55 kDa in the kidney [77]. Physiologically, UTs function to be responsible for urea transportation to maintain its concentration, cell homeostasis, and nitrogen balance. Moreover, UTs are important regulators of male reproductive function, blood pressure, bone metabolism, and brain astrocyte and cardiac functions. However, the available studies on the action of UT-B in the pathogenesis of varying diseases remain highly descriptive, and the functional mechanisms of UT-B currently remain elusive. Here, we provide a brief summary of the known functions of UT-B.

**Table 1:** Mammalian urea transporter gene

Gene	Chromosome	Isoform	RNA (kb)	Protein (kDa)	Cloned from	Tissue location	Inhibitors	References
Slc14a1	18 q12.1-q21.2	UT-B1	3.8	43	Human, rabbit, rat, mouse, Xenopus	Erythrocytes, brain, lung, heart, pancreas, colon, small intestine, prostate, kidney, bladder, skeletal muscle, bone marrow, cochlea	Phloretin, dimethylurea, acrylamide, methylurea, thiourea, methylformamide, PCMBS	[5, 55, 66, 92]
		UT-B2	3.7	43–54	Sheep, cow	Rumen		[66, 80]
Slc14a2	18 q12.1-q21.2	UT-A1	4.0	97,117	Human, rabbit, rat	Inner medullary collecting duct		[3, 68]
		UT-A1b	3.5	55		Medulla		[2]
		UT-A2	2.9			Thin descending limb, liver		[57, 73, 94]
		UT-A2b	2.5	44, 67		Medulla, heart		[2, 15, 31]
		UT-A3	2.1			Inner medullary collecting duct		[31, 32, 69]
		UT-A3b	3.7	43		Medulla		[2]
		UT-A4	2.5		Rat*	Medulla		[31]
		UT-A5	1.4		Mouse**	Testis		[19]
		UT-A6	1.8		Human***	Colon		[73]

*Cloned from rat only

^**^Cloned from mouse only

^***^Cloned from human only

## UT-B and the Kidd blood group

The Kidd blood group antigen Jk and UT-B are the same protein in erythrocytes [56]. In 1951, Mrs. Kidd gave birth to a newborn with hemolytic disease. The infant developed antibodies against a new blood group antigen, Jka, leading to the Kidd blood group [1] and its specific antibodies were identified later [61]. Currently, there are three antigens, Jka, Jkb, and Jk3, and four phenotypes, Jk (a+b-), Jk (a-b+), Jk (a+b+), and Jk (a-b-) in the Kidd blood group.

Although the Jk3-related JK (a-b-), an invalid phenotype, is rare, it is crucial for the safety of blood transfusion in humans because JK (a-b-) red blood cells are 30 times insensitive to urea lysis [28, 60]. The Jk-null phenotype is prevalent in Polynesian population, including Thailand (0.02%), Japan (0.002%), Taiwan (0.023%), China (0.008%), Chinese Han (0.019%), and Finland (0.03%) [39]. Genetic analysis indicates that the gene for Jk is located in chromosome 18q12-q21 [22] and encodes a 45-kDa glycoprotein UT-B with UT function [56, 71]. The JK-null phenotype is caused by the mutations in the UT-B splicing sites, and JK-null individuals have no functional UT-B protein, leading to a weaker urine concentrating ability. However, the absence of UT-B does not disrupt their homeostatic balance. Given the different sensitivities in gene mutation among different races, this steady state of homeostasis may not be applicable to all species. For example, aquaporin 1 (AQP1) knockout causes a severe defect in urinary concentrating in mice, whereas AQP1 deficiency does not lead to any pathological phenotype in humans. Thus, further studies are required to determine the roles of UT-B in homeostasis [48, 62, 67, 72].

The Kidd blood group is important for the safety of blood transfusion because its incompatibility can cause typical acute and delayed hemolytic transfusion reactions in newborns. According to the edition of the American Association of Blood Banks Technical Manual, there are anti-globulin or enzyme tests for the detection of Kidd antigens. The urea dissolution test for screening Jk (a-b-) phenotype is also available in America, especially in Hawaii [39, 41].

## UT-B proteins

The SLC14a1 for human UT-B was first cloned from human erythrocytes and is located at the single-gene locus of chromosome 18q12.1-q21.2 [77]. It is homologous to mouse [18]. There are two SLC14a1 mRNA transcripts due to variable polyadenylation, and they are expressed for a 45-kDa protein [44]. The UT-B-mediated urea transportation is inhibited by urea analogues and other compounds, including phloretin, dimethylurea, acrylamide, methylurea, thiourea, methylformamide, and PCMBS [52].

In humans, the SLC14a1 for UT-B is not only expressed in endothelial cell membrane of the renal medulla but also in erythrocytes and other organs, including the brain, lung, heart, pancreas, colon, small intestine, prostate, kidney, bladder, skeletal muscle, bone marrow, and cochlea [79]. The wide distribution of UT-B suggests that UT-B has broad physiological functions in humans.

UT-B in human body

### The urinary system

UT-B is detected in the renal epithelial cells, the ureter, and the bladder of rats. Similarly, UT-B is expressed in renal medullary descending vasa recta of mice [74]. Interestingly, genome-wide association study (GWAS) suggests that the UT-B polymorphism may be associated with the development of bladder cancer in Northern India.

The bladder can transport and store urine in mammals. High-protein diets can increase urea concentration, which may be a carcinogen for rat bladder [42, 70]. UT-B-/- mice were generated by disrupting the UT-B gene, evidenced by PCR analysis and lack of UT-B protein expression in all organs [86]. Transmission electron microscopy (TEM) analysis reveals that medullary sheath is observed in urothelial cells of UT-B-/- mice, which may cause urothelial cell shrinking, chromatin condensation, and apoptosis [91]. Microarray assay indicates that UTB knockout alters the expression of 69 genes associated with apoptosis and DNA damage in the urothelium of mice [13]. Among them, the DDB1- and CUL4-related factors 11 (Dcaf11) and MCM2-4 expression were significantly upregulated, while the ubiquitin carboxyl terminal hydrolase L1 (Uch-L1), adenovirus E1B-19K/Bcl-2 interaction protein 3 (Bnip3), and 45S pre-rRNA were downregulated. The frequency of UT-B efficiency is significantly higher than that of other transporters. Given that urea concentrations in the bladder of UT-B-/- mice are several times higher than those of wild-type mice, they can induce cell cycle arrest and apoptosis [36, 72, 92]. In the urothelium of UT-B-/- bladder, the decreased BcL-2 expression and increased caspase-3 and Bax expression, together with downregulated ubiquitin carboxy-terminal hydrolase L1 (UCH-L1), which promotes cell proliferation and enhances anti-apoptotic signaling pathway, may underlie the mechanisms by which high concentrations of urea cause urothelial cell apoptosis.

**Fig. 1:**
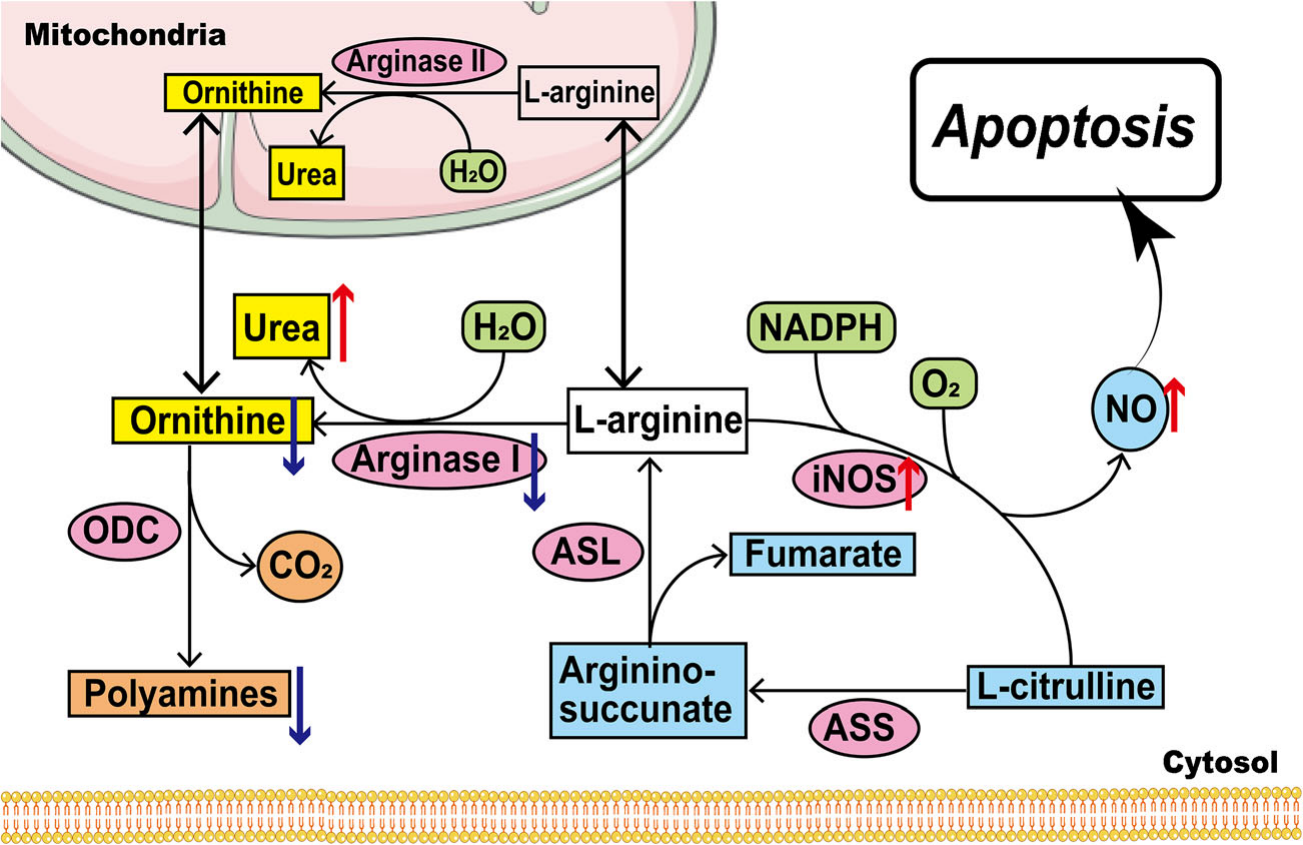
Arginine metabolism and the urea/L-Arg/NO pathway in urothelial cell apoptosis. NADPH, nicotinamide adenine dinucleotide phosphate; NO, nitric oxide; iNOS, inducible nitric oxide synthase; ASL, argininosuccinate lyase; ASS, argininosuccinate synthase; NO, nitric oxide; NOS, nitric oxide synthase; ODC, ornithine decarboxylase

L-arginine is cleaved by arginase to produce urea and ornithine, which can be preferably converted into polyamines by ornithine decarboxylase (ODC). Interestingly, the arginase I expression is significantly downregulated in urothelial cells of UT-B-/- mice [92]. This will reduce the production of ornithine and polyamine. Previous studies have shown that polyamines, especially spermine, are responsible for regulating gene expression and maintaining the stability of DNA and chromatin [32, 33]. On the other hand, high levels of nitric oxide (NO) are detected in the bladder and urinary epithelium of UT-B-/- mice, accompanied by increased iNOS expression [91]. Actually, high concentrations of NO can cause DNA damage and cell death in NO-generating and neighboring cells [47]. Hence, UT-B deficiency can increase urea concentrations, which cause abnormal arginine metabolism and increased levels of NO, leading to DNA damage and cell apoptosis (Fig. 1).

Alternatively, UT-B deficiency can enhance histone (H2AX) phosphorylation, but downregulate MCM2 expression. Given that H2AX is the indicator of DNA damage and MCM2 is an important component of MCM2-7 complex for DNA repair, their changes may also contribute to urea-induced epithelial cell apoptosis [91]. Furthermore, ataxia telangiectasia–mutated (ATM) kinase phosphorylation at SER1981 and p53 expression and phosphorylation at SER15 are significantly increased in bladder urothelium of UT-B-/- mice. These results suggest that DNA damage and apoptosis caused by the UT-B deficiency in the bladder urothelium may depend on the ATM/p53 signaling.

### The reproductive system

UT-B is also expressed in the testis, mainly in Sertoli cells of the seminiferous tubules at stages II to III [20]. Sertoli cells in the seminiferous tubules function to nurture the growing sperm cells during spermatogenesis. Similarly, high arginase activity can hydrolyze arginine into urea and ornithine in Sertoli cells and high concentrations of urea appear in testicular tissue of UT-B-/- mice, particularly for aging mice. Furthermore, UT-B-/- mice had higher body weights than wild-type mice, and earlier spermatogenesis and reproductive system maturation. However, there is no abnormality in the integrity of spermatogenic epithelial cells, sperm morphology or distribution, and the tubular and lumen diameters of the spermatogenic tubules in UT-B-/- mice [92].

It is well known that follicle-stimulating hormone receptor (FSHR) and androgen-binding protein (ABP) are crucial for the development and function of Sertoli cells in the testis [37]. FSH can through its FSHR stimulates ABP expression in Sertoli cells, which is important for the maintenance of sperm number. Actually, significantly higher levels of FSHR and ABP expression are detected in the testis of UT-B-/- mice at 10 days of age, accompanied by accelerating sperm formation and reproductive system maturation [92]. These suggest that UT-B-related urea concentrations may contribute to the development of Sertoli cells in the reproductive system of mice [25, 91].

### The circulatory system

UT-B is expressed in the heart. Electrocardiogram (ECG) indicated that P-R interval of adult UT-B-/- mice was significantly prolonged, and II-III grades of atrioventricular block appeared in elder UT-B-/- mice [53]. Further proteomic analysis reveals that nine protein expression is upregulated and one is downregulated in the heart of UT-B-/- mice and those upregulated proteins include troponin C (TNNC), troponin T2 (TNNT2) and troponin I (TNNI), desmin (DESM), acyl-CoA dehydrogenase short-chain (ACAD) precursor K7, K14, enolase (ENOA), malate dehydrogenase (MDH), and atrial natriuretic peptide (ANP). All of these proteins are associated with cardiac function, cellular energy metabolism, ion channel function, and oxidative stress [58, 84, 92, 95]. Interestingly, the levels of ANP expression in aged UT-B-/- mice are significantly higher than those in aged wild-type mice. ANP is expressed in the atrium and can regulate blood pressure, cardiac function and cardiovascular diseases, such as myocardial hypertrophy [65].

Du et al. [14] have shown that UT-B-/- mice are prone to cardiac oxidative stress and myocardial hypertrophy, accompanied by increased concentrations of urea in cardiac myocytes, and abnormal sugar and lipid metabolism. In addition, UT-B-/- mice display impaired ATP production, increased ROS production, and DNA mutations, which may result in mitochondrial dysfunction. These findings suggest that UT-B deficiency may impair myocardial energy metabolism and induce abnormal intracellular homeostasis. It is possible that UT-B-related urea concentration may affect the arginine-eNOS-NO pathway and blood pressure, leading to vascular relaxation [81].

### Erythrocytes

**Fig. 2:**
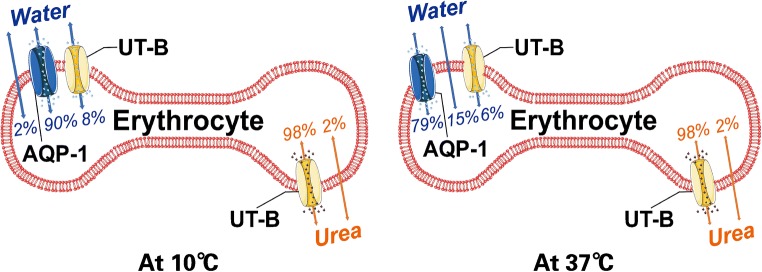
Contribution of AQP1, UT-B, and lipid bilayers to water and urea transport in erythrocytes. The transfer function of UT-B is regulated by temperature

UT-B protein is highly expressed in the plasma membrane of erythrocytes and is a water channel responsible for 8% of water transportation (at 10 °C). Although urea is not charged it has a strong dipole moment, which makes it impossible to penetrate through non-polar lipid membranes. Hence, urea depends on its specific transporters to pass through the cell membranes. The transfer function of UT-B is regulated by temperature (Fig. 2) [89, 90]. These, together with high permeability to urea in human erythrocyte, reduce urea concentrations in erythrocytes. Actually, UT-B deficiency decreases the urea permeability of erythrocytes by 45 times [91].

Physiologically, UT-B functions to protect erythrocytes from repeated osmotic pressure-related injury to maintain the stability of erythrocyte permeation and morphology. Because of higher concentrations of urea in the vasa recta higher permeability of AQP1 to water, it is crucial for the balance of transporting water and urea to avoid the high urea concentrations that damage erythrocytes in the renal medulla [21, 49]. Furthermore, rapid urea transport mediated by UT-B in erythrocyte may help establish urea concentration gradient. Erythrocytes can provide urea through UT-B for countercurrent exchange between ascending and descending rectal vessels.

### The bone marrow

The gene for UT-B was cloned from human bone marrow [55] and UT-B may influence bone metabolism [63]. Osteoporosis and senile osteopenia are characterized by reduced bone tissues and increased adipose tissues in the bone marrow. Given that adipocytes and osteoblasts are from mesenchymal stem cells, they can transdifferentiate in some conditions, such as aging, post-menopause, emergencies, and others to regulate the balance of hematopoiesis and osteogenesis in the bone marrow.

A previous study has shown that UT-B expression is downregulated during the adipogenesis in the bone marrow and nuclear hormone receptor peroxisome proliferator–activated receptor gamma 2 (PPARg2) expression is upregulated. Downregulation of UT-B occurred early before the increase of PPARg2 expression. The PPARg2 expression in human trabecular bone (hOBs) is upregulated during fat formation in hOBs. And there is a reciprocal relationship between the expression of UT-B and PPARg2; the time-dependent regulation of these two is opposite [63]. These findings suggest that UT-B-related urea concentrations in the bone marrow may regulate the differentiation of mesenchymal stem cells. During the urea cycle, arginine can be converted into urea or NO and polyamines. NO is an important mediator of osteoblast activity and a stimulator for bone formation. NO can regulate the biochemical processes in chondrocytes, osteoblasts, and adipocytes [9, 24, 50]. Polyamines are polycations and can interact with negatively charged DNA, RNA, and proteins to regulate the proliferation and differentiation of many types of cells. Therefore, UT-B expression in osteoblasts may be a marker of fat formation in the bone marrow during the process of osteoporosis and also provide theoretical basis for understanding the mechanism underlying osteoblasts/adipocytes differentiation [63].

### The digestive system

UT-B2 mRNA transcripts can be detected in the rumen of sheep and cow [11] and its expression is regulated by gastric acidity and alkalinity, rumen ammonia concentration, and the type of food consumed in the digestive system of sheep and cow. For example, consumed solid food can increase UT-B expression in cows [4] and feeding with a large amount of nitrogen and non-fiber carbohydrates also upregulates UT-B expression in the rumen of sheep [43]. Furthermore, short-chain fatty acids and low pH can increase the UT-B protein expression in rumen epithelial cells of sheep. Similarly, several factors can regulate UT-B2 expression in ruminants of rodents.

**Fig. 3:**
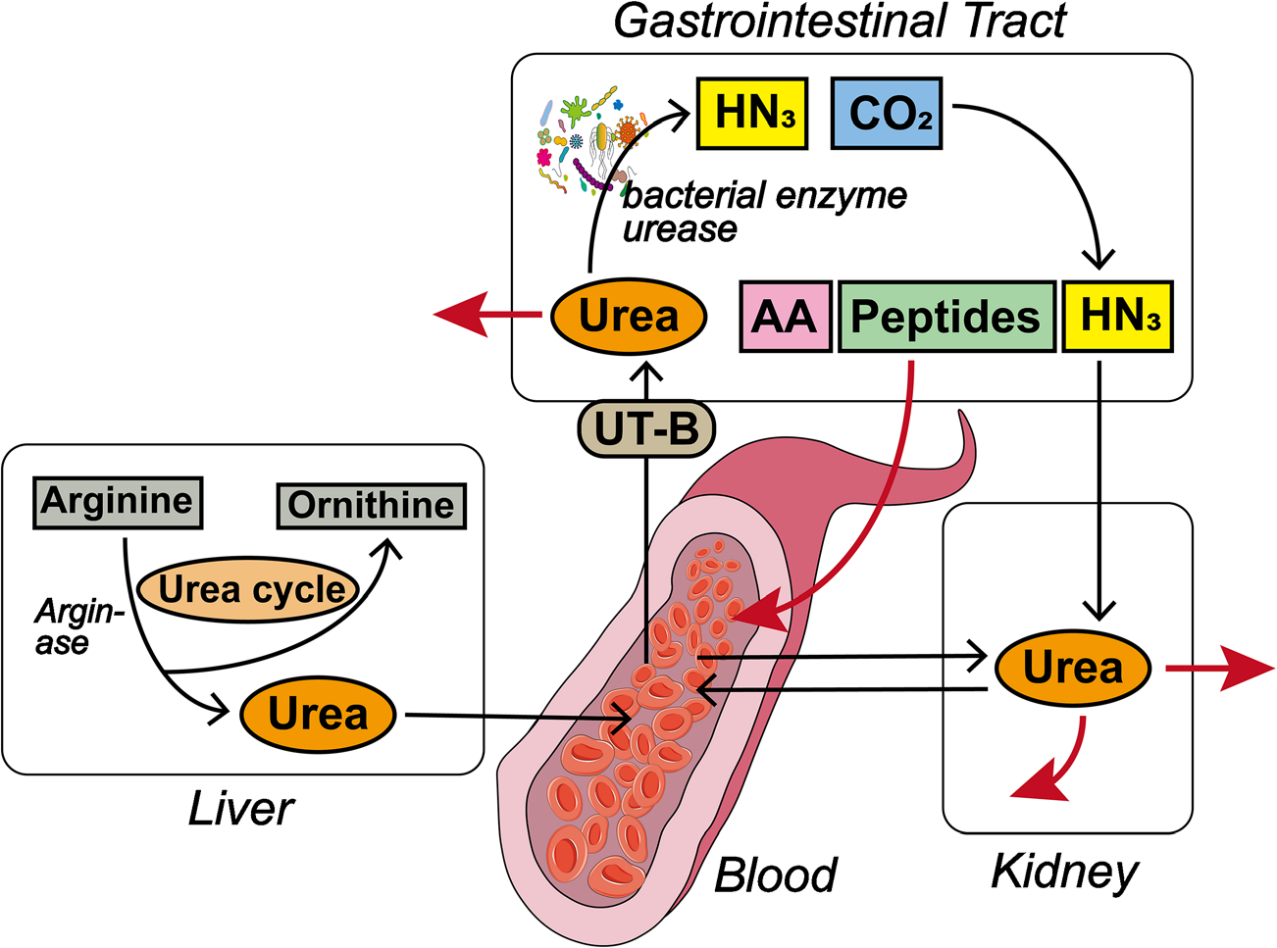
The urea nitrogen salvaging (UNS) process. Urea is produced in the liver via the urea cycle and enters into the blood. There are two main destinations for urea produced in the liver: (a) Pass through the kidney and (b) pass through the gastrointestinal tract via UT-B. Urea entering the kidney can be freely filtered and reabsorbed or excreted directly. The gastrointestinal tract contains a large number of bacteria, and the gastrointestinal urea is decomposed into ammonia and carbon dioxide by bacterial urease. The ammonia can be directly absorbed by the blood or be used by bacteria to produce amino acids (AA) and peptides that are reabsorbed

More importantly, UT-B is also expressed in human digestive system. Firstly, UT-B protein is detected in human small intestine, colon, and colonic recesses [64]. Furthermore, UT-B protein is detected in human intestinal Caco-2 cells and epithelial cells, particularly higher expression in ascending colon than in descending colon [10, 86]. UT-B has shown to promote the transportation of urea from blood to the gastrointestinal tract in the ruminants and other mammals [45, 51, 83] and the gastrointestinal UT-B in humans may participate in the process of urea nitrogen salvaging (UNS). During the UNS process, urea supplied by animals is decomposed by bacterial urease. Amino acids and peptides released by bacteria are absorbed through the transport system of epithelial cells to help the body growth and also to maintain a healthy microflora in the intestine (Fig. 3) [78]. The microbial population in human gastrointestinal tract varies greatly and is associated with the development of many diseases, such as diabetes, inflammatory bowel disease, and obesity [12, 16, 17, 34].

### The central nervous system

Urea can regulate the contents of ammonia and nitrogen. These polar molecules are mainly formed in the liver through the urea cycle. However, the urea cycle in the CNS is incomplete although the levels of urea are similar to that in the liver [5].

UT-B is important for the maintenance of urea concentrations in the CNS. Firstly, UT-B is expressed in the olfactory bulb, cortex, caudate nucleus, hippocampus, and hypothalamus of mice [40]. Furthermore, UT-B is also expressed by astrocytes as UT-B is co-localized with GFAP, a marker of astrocyte, in the brain sections. However, there is no evidence of UT-B expression in oligodendrocytes, microglia, and vascular endothelial cells. In addition, UT-B-/- mice display depressive-like behaviors [5]. This outcome may stem from UT-B deficiency-increased urea contents in the cerebral cortex and hypothalamus and lower NO in the hippocampus of mice. In the chronic mild stress animal model that mimics human depression, antidepressant fluoxetine inhibits NO production in the hippocampus [46]. Actually, urea loading can reduce NO production in the hippocampus of UT-B-/- and UT-B-/+ mice. UT-B deficiency does not alter NO concentrations in blood, the cortex, and hypothalamus although it increases nNOS, but not eNOS and iNOS expression in the hippocampus of mice. In addition, nNOS reduces 5-HT transporter activity, which feedback decreases the 5-HT uptake–stimulated nNOS activity and NO production [8, 23]. In a uremic condition, urea can competitively inhibit the transport of L-arginine (L-arg) to endothelial cells through the UT-B, which can be eliminated by urea transport inhibitors [92]. These observations support the notion that high urea concentrations can reduce NO level, but enhance nNOS expression [85, 88, 96]. The decrease in NO content may be related to the competition of arginine with nNOS for the NO substrate L-arginine, which needs to be further investigated. Furthermore, the reduced NO by UT-B deficiency subsequently decreases cerebral blood flow (rCBF) [82] and increases neuronal degeneration, vacuolar, and myelinated and unmyelinated fiber formation, which may contribute to the development of depression in mice.

UT-B may be crucial for the balance of sodium and water in the cerebrospinal fluid (CSF). Actually, long-term feeding with high salt diet not only significantly elevated [Na+] in the CSF but also significantly reduced UT-B, but not UT-A, expression in epithelial cells of the choroid plexus (CP) in salt-sensitive Dahl S rats, indicating that increased [Na+] in the CSF was associated with the high urea concentration caused by the downregulated UT-B expression in the CP. It is possible that the high Na+ concentrations in the CSF induced by high salt diet may increase arginine vasopressin levels to decrease UT-B expression in the Dahl S rats [26].

### The cochlea

Western blot and immunohistochemistry reveal that UT-B is expressed in pillar, hair, and Boettcher’s cells of both the inner and outer ears [38]. The inner ear is responsible for sound and maintaining balance. It is well known that urea is a marker for diagnosis of too much endolymphatic effusion. The contents of urea and glycerol are commonly used for diagnosis of Meniere’s disease. Urea can increase the permeability gradient between blood and inner ear fluid and reduce the volume of endolymph hydrops [7, 30, 87]. The steady state of the volume, pressure, and chemical composition in the endolymph is crucial for the electromechanical conduction of sound in the ear. However, urea administration can increase serum osmotic pressure and osmotic gradients between blood and inner ear fluids [17, 75, 93]. In the cochlea, the penetration of small hydrophilic solutes through the blood labyrinth barrier depends on the molecular weight of solutes. Because urea transport rate is much faster than some smaller hydrophilic molecular solutes, such as L-glucose, mannitol, and sucrose, and glycine [26, 76], the urea transport system is important for the homeostasis of the volume, pressure, and chemical composition in the endolymph of the cochlea. Coincidentally, UT-B is expressed in the supporting cell system of the cochlea, such as Boettcher’s cells, which can contact with endolymph, reticular layer, and perilymph. UT-B expression in these cells may be important for the urea transport between endolymph and perilymph.

This review provides a systematic overview of the physiological function of UT-B. Currently, the available studies centered on UT-B deficiency-related phenotypic manifestations, but little is known on the precise mechanisms underlying the action of UT-B. Currently, there is no information on the exact factors that influence UT-B expression although some studies have shown that low protein diet and solid foods upregulate UT-B expression in the digestive system of rats and in the rumen of cow and sheep. UT-B is an N-linked glycosylated urea transport protein and is widely distributed in human body. Functionally, UT-B is responsible for intracellular and extracellular urea transport, maintaining urea concentrations.

Urea is the product of urea cycle (ornithine cycle), which is essential for life activities. UT-B knockout significantly increases urea concentrations in the bladder of mice and high concentrations of urea can feedback inhibit urea cycle and damage the structure and function of tissues and organs. Actually, UT-B deficiency caused DNA damage and apoptosis in bladder epithelial cells and the precocious reproductive system in mice. UTB deficiency can also lead to prolonged P-R interval and atrioventricular block in mice, accompanied by abnormal mitochondrial function and myocardial energy metabolism. Therefore, UT-B deficiency may be associated with the development of bladder cancer [29] and myocardial hypertrophy as well as other diseases.

The high urea concentrations caused by UT-B deletion can affect the NO/NOS system. NO is important for cardiovascular and nervous functions. In the CNS, NO participates in the process of sleep-wake cycles, neurosecretion, and reproduction. High levels of NO can convert into reactive nitrogen species (RNS), which can cause cell damage [6]. Interestingly, UT-B deficiency decreased the NO content in the hippocampus of mice with depressive behavior. Although the relationship between UT-B efficiency-reduced NO and depression remains to be determined this phenotype suggests that the UT-B-related NO pathway is involved in the unknown neuromechanism. In addition, NO may promote depression by regulating cerebral blood flow. Given that UT-B can regulate urea cycle by adjusting urea concentration and all enzymes related to urea cycle are expressed in the brains of AD individuals [27], it is unclear whether the altered urea cycle by UT-B deficiency contributes to the pathogenesis of AD.

The potential clinical application of UT-B is enormous. Currently, studies have shown that UT-B reduces the apparent diffusion coefficient of ^13^C hyperpolarized urea in tissues by mediating the uptake of urea by cells, suggesting that UT-B has the potential to become a magnetic resonance–based gene reporter in vivo [59]. In addition, UT-B inhibitors are being developed for diuretic targeting and may overcome the side effects, such as hypernatremia of conventional diuretics because UT-B inhibitors usually do not change serum sodium, chlorine or potassium levels. Therefore, UT-B inhibitors may be particularly useful in reducing circulation volume in patients with congestive heart failure [35].

In summary, future researches on UT-B should investigate whether UT-B can directly regulate organ function besides maintaining urea concentration, and which factors can modulate UT-B expression. To address these questions may provide new insights into the physiological action of UT-B and aid in design of new therapeutic strategies for treatment of relevant diseases in the clinic.
